# Meteorology
*Meteorology. By James Glaisher, F.R.S., F.R.A.S. Excerpt paper from Hughes's Reading Lesson Books. London: Longmans. 1857.


**Published:** 1857-04

**Authors:** 


### METEOROLOGY.*

Our meteorologists will be glad to know that Mr. Glaisher has brought out, in Hughes's series, a short but excellent, and, what is more, cheap, treatise on their science. He describes the use of the barometer, the vernier, the thermometer, and the hygrometer. He supplies chapters on vapour, rain, snow, and ozone; illustrates his pages with nicely executed drawings of clouds, and snow crystals; and finishes with an useful series of tables. The book is so easily and scientifically written, that child and man may learn from it with equal pleasure and profit.

* Meteorology. By JAMES GLAISHER, F.R.S., F.R.A.S. Excerpt paper from Hughes's Reading Lesson Books. London: Longmans. 1857.

